# The “Tetris effect”: autistic and non-autistic people share an implicit drive for perceptual cohesion

**DOI:** 10.1186/s13229-025-00654-4

**Published:** 2025-03-26

**Authors:** Nazia Jassim, Brónagh McCoy, Esther Wing-Chi Yip, Carrie Allison, Simon Baron-Cohen, Rebecca P. Lawson

**Affiliations:** 1 Department of Psychology, University of Cambridge, Cambridge, UK; 2 Department of Psychiatry, Autism Research Centre, University of Cambridge, Cambridge, UK

**Keywords:** Visual perception, Tetris, Perceptual cohesion, Spatial cognition, Attention, Autism, Neurodiversity, Replication, Open materials

## Abstract

**Background:**

When working on jigsaw puzzles, we mentally “combine” two pieces to form a composite image even before physically fitting them together. This happens when the separate pieces could logically create a cohesive picture and not when they are mismatched or incoherent. The capacity of the brain to combine individual elements to form possible wholes serves as the basis of perceptual organisation. This drive for perceptual cohesion—the “Tetris effect”—can be seen in the famous game, where people automatically perceive logical combinations from separate pieces. However, it is unclear how this presents in populations known to have perceptual differences, such as autistic people. Theories on the inclination to process local over global details in autism and autistic strengths in pattern recognition lead to conflicting predictions regarding the drive for perceptual cohesion in autistic compared to non-autistic people.

**Methods:**

In this large-scale (*n* = 470) pre-registered online behavioural study, we aimed to replicate previous research conducted on neurotypical participants and to extend this work to autistic participants. We used two tasks with Tetris-style stimuli to examine how autistic (*n* = 196) and non-autistic (*n* = 274) adults implicitly perceive possible wholes from individual parts. Data were analysed using logistic mixed-effects regression models and hierarchical Signal Detection Theory modelling.

**Results:**

Overall, we replicated the results from the original study in finding participants are more likely to perceive parts as wholes when there is the potential to form a whole, compared to when there is not. However, we found no differences between autistic and non-autistic participants across both tasks.

**Limitations:**

Although power calculations were carried out to assess sample sizes needed to detect a group difference, given the small effect size (Cohen’s d = 0.37) in the original study, it may be that any existing group differences are still undetectable with the current sample size.

**Conclusions:**

We conclude that the “Tetris effect” is ubiquitous and seen in both neurotypical and neurodiverse populations. Our findings challenge the deficit-focussed narrative often seen in the autism literature and highlight the similarities in task performance between autistic and non-autistic participants.

**Supplementary Information:**

The online version contains supplementary material available at 10.1186/s13229-025-00654-4.

## Background

Our brains have the remarkable ability to construct cohesive scenes from disjointed parts. This is commonly seen during classic games such as jigsaw puzzles, Lego, and Tetris. Here we tend to perceive possible logical combinations even before the individual pieces come together.

For more than a century now, psychologists have been exploring how our perceptual systems organise or group separate elements into composite “wholes”. Gestalt psychologists proposed that this is governed by simple laws of perceptual organisation [1]. They further posited that perceptual organisation is automatic and involuntary, and occurs through spontaneous processes in the brain. Hermann von Helmholtz proposed that the visual system unconsciously chooses the most likely interpretation of the composite “whole” based on the distal layout; this may also lead to automatically “filling in” missing information [2]. More recent Bayesian theories suggest that, in the absence of perspective cues, humans tend to rely on prior knowledge to guide visual perception [3, 4].

The concepts surrounding perceptual organisation may have particular relevance for certain populations, notably autistic individuals. Autism encompasses a set of neurodevelopmental conditions characterised by difficulties in social interactions and verbal and nonverbal communication; unusually narrow interests; restricted, repetitive behaviours; and sensory-perceptual disturbances [5, 6]. Autistic people are hypothesised to have “detail-focused” perception or “Weak Central Coherence” due to a bias for local details at the expense of the global picture [7]. A different account sees autistic people’s superior attention to detail as in the service of “hyper-systemizing”, defined as the drive to analyse or construct systems or rule-based patterns [8]. It has been proposed that autistic people rely less on prior knowledge during visual perception, making their perception comparatively less “top-down” or conceptually-driven [9]. As a result of this reduced reliance on prior knowledge, they are hypothesised to have a “more accurate”, sensory data-driven perception [9].

Visual perception research in autism has predominantly concentrated on the trade-off between local and global processing, with task performance being attributed to one or the other [10, 11]. According to the “global precedence hypothesis”, neurotypical individuals first process the global properties of a visual scene, followed by the more fine-tuned, local features [12]. In contrast to this, autistic children have been found to show local-to-global precedence during scene perception [13]. This may explain the autistic advantage in experiments where local-to-global precedence may prove beneficial, such as figure dis-embedding and identifying hidden targets in complex scenes [14]. Autistic individuals were found to outperform non-autistic controls in the mental segmentation component of the Block Design Test, which assesses the ability to mentally segment and rearrange blocks to create patterns of increasing difficulty [15]. However, more recent work has suggested that autistic individuals may be *slower* to process global wholes [16], or may merely be less inclined to use global-to-local processing strategies during visuospatial tasks [17]. In line with this, autistic individuals have been found to differ in their response strategies during two alternative forced-choice figure dis-embedding tasks [18]. To minimise the role of explicit cognitive strategies, we propose the use of indirect measurements may better capture the automatic and implicit aspects of perception in autism.

Contrary to the predictions of local-to-global precedence and bottom-up theories of autistic perception, autistic individuals have been found to show either superior or comparable performance to neurotypical controls in visuospatial reasoning, often in tasks that require switching between local and global processing, high-level mental imagery, and top-down processing [10, 19]. A real-world example of this is seen in building blocks or Lego, a game which is gaining increasing significance for its therapeutic benefits in supporting autistic children in their social communication development [20]. Conducting fundamental research on how autistic people perceive and process the raw (digital) materials used in play-based interventions such as Lego and its modern counterpart, Minecraft, may help to understand the mechanisms by which these therapies can help autistic people, whether through sensory perception or other cognitive domains like executive functioning. It is likely that autistic individuals simply have a higher drive to “systemize” or identify patterns or rules in a mechanical system [8]. However, it is unclear whether this is a function of high-level cognition or arises automatically and implicitly at the level of low-level perception. For example, certain visuospatial reasoning tasks, including pattern-matching and mental rotation, have been found to evoke mental imagery at an unconscious—rather than conscious—level [21]. One explanation is that the “need for sameness” associated with autism may be connected to a need for perceptual cohesion in the sensory environment. Examining how autistic people implicitly perceive cohesive structures from separate pieces may provide fresh insights into perceptual organisation in autism.

In this study, we employ simple, familiar stimuli to examine how autistic and non-autistic adults implicitly integrate structural pairs of stimuli to form cohesive wholes. To this end, we implement Tetris-style stimuli and experiments previously developed and reported by Guan and Firestone (2020) [22]. In the original study, they found evidence to suggest that neurotypical individuals confuse disjointed parts for their potential wholes. In other words, when two puzzle pieces can logically fit together to create a cohesive whole, individuals tend to involuntarily perceive the separate pieces as a composite whole. Here we aim to a) replicate findings from the original study [22] and b) examine how this “Tetris effect” presents in autistic compared to non-autistic participants. We propose that the combination of implicit processing and gamified stimuli may reveal novel insights about autistic perception.

## Methods

### Participants

A total of 470 individuals aged 18 years and above, with or without autism diagnoses, and with normal or corrected-to-normal vision participated in the study. The participant groups comprised 196 autistic individuals and 274 non-autistic control participants. Demographic and clinical information can be found in Table 1. We report key co-occurring conditions, specifically ADHD and mental health conditions, given their high prevalence of co-occurrence with autism [23, 24].

**Table 1:** Demographic and clinical information

Groups	N	N female	Age (years)	AQ	STAI- trait	Other diagnoses (%)		
						Anxiety disorders	Mood disorders	ADHD
Autistic	196	120	41.1 (12.33)	32.4 (4.30)	54.7 (12.3)	87	114	38
Control	274	170	35.5 (12.95)	20.9 (7.60)	46.0 (13.83)	66	70	11

AQ and STAI questionnaires were not completed by the full sample but were collected for 186 autistic participants and 233 control participants

### Procedure

The stimuli and experiments used in this study were originally developed and reported by Guan & Firestone (2020) [22].

### Sample size estimation

The study sample size was based on a power analysis on results of the first experiment reported in the original study [22]. The original result was based on a paired-samples *t*-test between *potential* and *no potential* trials*.* To calculate the minimum sample size necessary to detect the “Tetris effect” in each group, and replicate this result, we used a Cohen’s *d* effect size of 0.37, as reported in the original study [22], a significance level of *p* < 0.001, and a desired power of 80%. This yielded a minimum sample size of *n* = 130 per group. As we were interested in detecting a group difference we also carried out a separate two-sample power analysis, with a Cohen’s *d* effect size of 0.3 (to detect at least a small effect size), a significance level of *p* < 0.05, and a minimum power of 80%. This resulted in a minimum sample size of *n* = 175 in each group. We therefore aimed to collect a minimum of *n* = 175 participants in each group to address both of our research questions.

### Data collection

Data collection was done over two phases using online behavioural research platforms. In Phase 1, participants were recruited via social media and email notifications sent to the Cambridge Autism Research Database (CARD). The experiments were built using the online experiment-builder lab.js [25] and embedded into Qualtrics, an online platform for surveys. In Phase 1, 296 participants (188 autistic individuals, and 108 non-autistic controls) took part in the study. Due to technical issues with Qualtrics, namely an update imposing a restriction on embedded data length, task data could no longer be collected via this platform. Phase 2 of data collection was primarily established to bolster the sample size from Phase 1. In Phase 2, 174 participants (8 autistic, and 166 non-autistic controls) were recruited via Prolific, an online recruitment platform, and directed to complete the study on Gorilla, a web-based platform for behavioural experiments [26]. The data collection phase was accounted for in all our analyses unless otherwise stated. Participants were asked to confirm official diagnoses by choosing from a drop-down list of mental health and cognitive conditions. Those who indicated an autism diagnosis were further asked to confirm which professional diagnosed them: GP, psychologist, psychiatrist, neurologist, or “other”. All 470 participants were required to complete the same questionnaires and behavioural experiments.

### Questionnaires

In addition to the behavioural experiments, participants completed the following questionnaires that are of relevance to autism: 1) the short-version of the Autism Spectrum Quotient (AQ-10) [27], and 2) the State-Trait Anxiety Inventory (STAI) [28, 29]. Consisting of 10 items, the AQ-10 is a brief version of the 50-item Autism Spectrum Quotient [30] and is recommended by the National Institute for Health and Care Excellence (NICE) as a screening tool for autism in adults. The AQ-10 is used to measure autistic traits in the general population and is not considered a diagnostic instrument. The STAI is a 40-item inventory to assess individuals’ anxiety across state and trait domains [29]. In addition to autistic traits, we also measured participants’ trait anxiety, as anxiety and autism often overlap, with some aspects appearing to be indistinguishable from one another [31]. We include autistic and anxious traits as additional independent variables in task regression analyses, to address transdiagnostic factors and tease out these effects from those due to a diagnosis of autism alone. See supplement for details about the questionnaire scoring and distributions of scores (Fig. S1).

### Task 1: Target detection

#### Stimuli and design

**Fig. 1:**
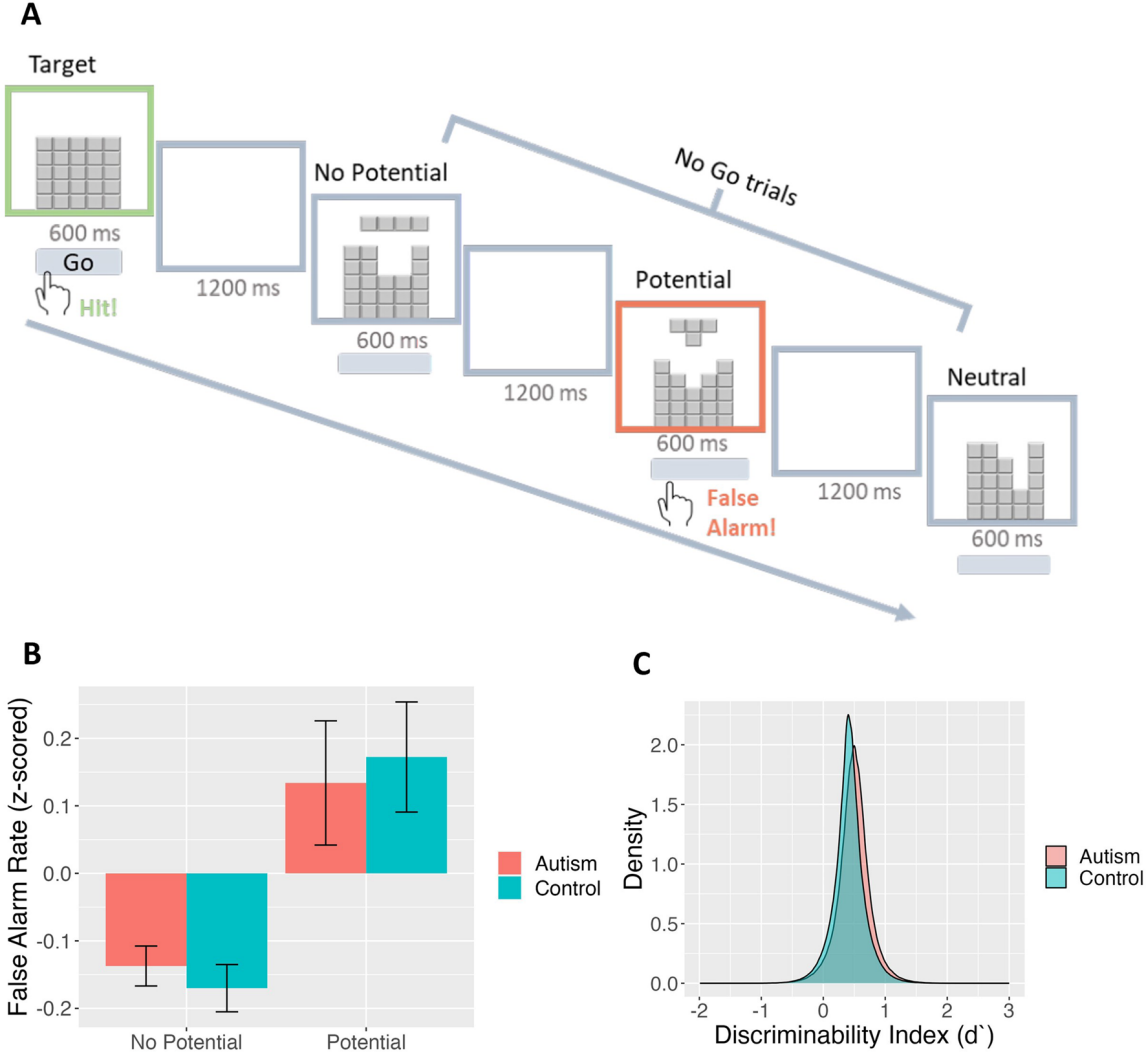
Target Detection task. **A** Target Detection consisted of a Go/No Go paradigm using Tetris-style stimuli developed by Guan and Firestone (2020) [22]. Stimuli were made up of “complete” blocks (Target/ Go), disjointed blocks (No Go), and neutral incomplete blocks (No Go). No Go trials containing disjoined blocks could be further categorized as potential (two disjointed blocks that could logically fit together to form a complete block) and no potential trials (two disjointed blocks that cannot be combined to form a coherent block). Participants were instructed to press a button in response to the target trials. Correct hits led to the border turning green for 200 ms, while false alarms led to a red border being displayed. Examples of correct and wrong trials are shown in the figure. The experiment consisted of 84 trials with each trial lasting 600 ms and separated by an inter trial interval of 1200 ms. **B** False Alarm Rates (z-scored). False alarms across all No Go trials (potential and no potential) and group (Autism in red and Control in blue) were first z-scored separately per experimental dataset. This figure shows the z-scored False Alarm Rate combined across datasets. Error bars represent ± 1 SEM C) Signal detection theory analysis results showing posterior distributions of the discriminability index (d’) values for Autism (in red) and Control (in blue) groups

This task was based on the classic Go/No Go paradigm in which participants were instructed to press a key in response to a frequently occurring target trial (i.e., Go trials) and to refrain from pressing the button during the less-frequent No Go trials [22]. The stimuli were made up of “Tetris”-style blocks. The target trials were identified as those containing a complete block, while the No Go trials belonged to 3 categories: *potential* trials consisting of two disjointed blocks that could seemingly combine to form a complete block; *no potential* trials consisting of two disjointed blocks with no possibility of combining to form a complete block; and neutral trials comprising a single incomplete block. Each trial lasted 600 ms with an inter-trial interval of 1200 ms. Participants were instructed to press the spacebar when presented with the target Go trials. Correct responses (i.e., responding to the target) were indicated by the border turning green, while incorrect responses (i.e., incorrectly responding to non-target stimuli or not responding to the target) led to a red border. The feedback was displayed for 200 ms. The task consisted of a total of 84 trials, with 24 repetitions of the target, *potential,* and *no potential* trials each, and 12 repetitions of the neutral trials. Participants completed a brief practice session of 5 trials prior to the main task. Trial order was randomised across participants. A schematic representation of the Target Detection task can be seen in Fig. 1.

#### Data analysis

Data were analysed using R version 4.3.1 [32]. Participants who scored below 50% accuracy on Go trials were excluded. Performance on Go trials was assessed primarily for attention checks, and the main analyses were limited to the No Go trials.

Here we tested the hypothesis that individuals may be more likely to false-alarm to *potential* No Go trials than *no potential* No Go trials. To assess the effects of No Go trial type on false alarm rates, we ran a series of logistic mixed-effects regression models using the ‘lme4’ R package [33]. To examine group differences in false alarms, we ran models using false alarm rate as the dependent variable and group (Autism vs Control) and No Go trial type (*potential* or *no potential*) as independent variables. Main and interaction effects were calculated. Each participant was included as a random factor. Age and sex assigned at birth were included as regressors in all models. As data were collected using different online research platforms over the two phases, we also included data collection phase as a regressor in all models. Additional models were run after replacing diagnostic groups with relevant questionnaire scores (Supplement). Finally, as autism often co-occurs with ADHD and anxiety, we also ran supplemental models using these diagnoses as regressors to rule out any possible confound.

Next, we implemented a Bayesian Signal Detection Theory (SDT) analysis in a hierarchical framework using the “hBayesDM” package in R [34]. The SDT analysis was not included in the original study [22]; we conducted this analysis to confirm findings from the regression models and to help understand underlying mechanisms. Here we focused on group differences in the sensitivity/discriminability index (*d′*), which assesses sensitivity to the difference between *potential* trials and *no potential* trials. See the supplement for further details about the SDT analysis. As the effect of the dataset could not be included in this model, we conducted SDT analyses separately on the Phase 1 and Phase 2 data to ensure that the direction of the results were consistent across both datasets (see Supplement). Finally, to examine whether the discriminability index (*d′*) was associated with autistic or anxious traits, derived from the AQ and STAI respectively, linear regressions were performed with each model parameter as the dependent variable and AQ, STAI, age, and sex as independent variables (see Supplement).

### Task 2: Colour identification

#### Stimuli and design

**Fig. 2:**
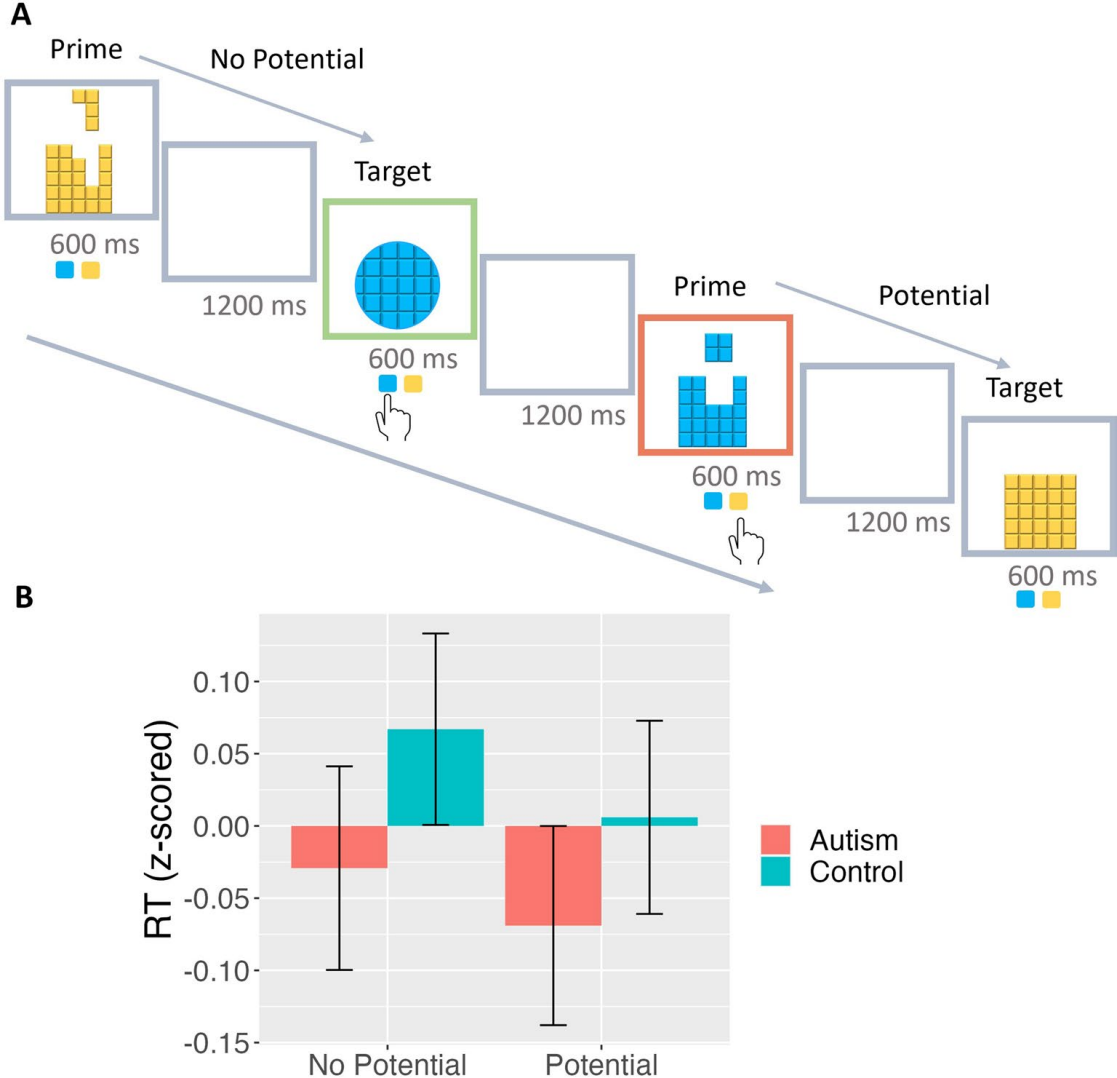
Colour Identification task. **A** Colour Identification task developed by Guan & Firestone (2020) [22]. The stimuli consisted of: a complete square, a complete circle, or a disjointed square or circle which may seem to fit together like puzzle pieces. The potential condition occurs when the previous trial (or the “prime” trial) may be a potential version of the subsequent target trial. In the no potential condition, the prime trial is a completely different shape to the subsequent trial. Participants were simply instructed to indicate the colour of the stimuli (either blue or yellow) by means of a button press. There were a total of 128 trials, each lasting 600 ms and separated by an inter-trial interval of 1200 ms. Correct responses led to the border turning green for 200 ms, while wrong responses led to a red border. Examples of correct and wrong trials are shown in the figure. **B** Colour Identification task results: RT distributions z-scored separately across target trials in each dataset (Autism in red and Control in blue). Error bars represent ± 1 SEM

To account for the possible effects of attention on shape perception, the Colour Identification task required participants to attend to the stimulus colour rather than shape [22]. Here the stimuli consisted of Tetris-style shapes which may be one the following: a complete square, a complete circle, or a disjointed square or circle which may seem to fit together like puzzle pieces. In this task, two subsequent trials could be categorised as either a *potential* or a *no potential* condition. The *potential* condition occurs when the previous trial (or the “prime” trial) may be a potential version of the subsequent target trial. In the *no potential* condition, the prime trial is a completely different shape to the subsequent trial; in other words, it cannot possibly combine to form the same shape as the target trial. Here we assess whether individuals could be primed to respond faster to the target when the prime trial is a possible disjointed version of the target. Hence reaction times (RT) to the target were our measurement of interest. *Potential* and *no potential* conditions were categorised only on the basis of shape and not colour. Participants were simply instructed to indicate the colour of the stimuli (either blue or yellow) by means of a button press. There were a total of 128 trials, each lasting 600 ms and separated by an inter-trial interval of 1200 ms. Correct responses led to the border turning green for 200 ms, while wrong responses led to a red border. Trial order was randomised across participants. A schematic representation of the Colour Identification task can be seen in Fig. 2*.*

#### Data analysis

Data were analysed using R version 4.3.1 [32]. Exclusions were based on task accuracy. Due to the relatively simple task instructions, participants who scored less than 80% correct were excluded from the analyses. Reaction times of less than 200 ms were excluded as outliers.

We then conducted a linear mixed-effects regression on RT using the R packages “lmer4” and “lmerTest” [33, 35]. We focused on correct responses for the RT analyses. We included target RT as the dependent variable, and group (Autism vs Control) and condition (*potential* vs *no potential*) as independent variables. Age, sex assigned at birth, and data collection were included as regressors in all models. We also controlled for the possible effects of stimulus shape (square or circle) and colour (blue or yellow) on RT. To examine possible effects of autistic or anxious traits on task performance, these models were run after replacing the diagnostic group with AQ and STAI scores (Supplement). Finally, to account for possible confounds, we ran supplemental models using ADHD and anxiety diagnoses as regressors.

## Results

### Task 1: Target detection

296 participants completed the task in Phase 1. For statistical purposes, five participants who did not provide information on sex assigned at birth were excluded from the analyses. We further excluded 4 participants who responded to the target less than 50% of the time. In Phase 2, out of the 174 participants, five participants did not provide information on sex assigned at birth and 2 participants responded to the target less than 50% of the time. Task data from a total of 454 (188 autistic, 266 controls) participants were analysed. Target detection accuracy was high in both groups: 96.14 ± 19.11% (Autism: 95.2% ± 21.3%, Control: 97.1% ± 16.9%), with no difference across groups according to a logistic-mixed effects model with group, age, gender, and data collection phase included as independent regressors (b = 0.179, z = 0.74, *p* = 0.459).

The overall false alarm rate was 0.92 ± 3.06%. A paired-samples t-test on false alarms confirmed findings from the original study [22], with participants making more false alarms on *potential* No Go trials than on *no potential* trials (t(584.17) = 4.70, *p* < 0.001). In other words, participants were more likely to false-alarm to stimuli showing two disjointed parts that had the *potential* to form the target, as opposed to the two disjointed parts that could not logically fit together to form the target (i.e. the *no potential* stimuli). A logistic mixed effects regression on false alarms was then carried out to assess any differences according to autism diagnosis, while also taking demographic and experimental information into account. This again confirmed a main effect of No Go trial type, with *potential* stimuli resulting in significantly more false alarms than *no potential* stimuli (*b* = 1.10, *z* = 5.49, *p* < 0.001; see Fig. 1). There were also significant effects of data collection phase (*b* = 0.96, *z* = 3.14, *p* = 0.002) and age (*b* = − 0.61, *z* = 4.42, *p* < 0.001), with more false alarms in general in the Phase 2 dataset and for younger participants. There was no effect of sex (*b* = 0.36, *z* = 1.48, *p* = 0.139). Importantly, there was no effect of autism diagnosis on false alarm rate (*b* = 0.11, *z* = 0.26, *p* = 0.793), nor any interaction of autism diagnosis and *potential* trial type (*b* = 0.28, *z* = 0.76, *p* = 0.446). Likewise, there were no main effects of AQ on false alarm rate (*b* = − 0.04, *z* = − 0.17, *p* = 0.862) (see Supplement).

To assess whether anxiety or ADHD—conditions that often co-occur with autism—obfuscated any effects of autism, we conducted a separate logistic mixed-effects regression with anxiety and ADHD diagnoses as additional regressors. This additional analysis confirmed the initial finding of no effect of autism (*b* = 0.17, *z* = 0.40, *p* = 0.692), and no interaction between autism diagnosis and *potential* stimuli (*b* = 0.28, *z* = 0.76, *p* = 0.446) on false alarms. Furthermore, there were no significant effects of anxiety (*b* = 0.11, *z* = 0.40, *p* = 0.686) or ADHD (*b* = − 0.77, *z* = − 1.58, *p* = 0.113) diagnoses on false alarms.

Next, separate Bayesian SDT models were conducted for the Autism and Control groups. Overall, all participants were similarly sensitive to the difference between *potential* and *no potential* stimuli (Autism: 0.50 ± 0.12, Control: 0.42 ± 0.08; 95% HDI = [− 0.21, 0.36]) (Fig. 2). The lack of group differences in the discriminability index (d′) is indicated by the highest density interval (HDI) of the difference in the group distributions overlapping 0. For completeness, an SDT model was also applied in a traditional approach on Go vs. No Go trials (see Supplement).

### Task 2: Colour identification

In Phase 1, 287 participants completed the Colour Identification task. Of these, we excluded eight participants who did not provide information about sex, and eight participants with accuracy rates of less than 80%. All participants’ RT were above the lower cutoff of 200 ms. We further excluded 14 participants whose mean RTs were extremely close to the timeout, with the same intraindividual RT for each condition, suggesting a technical error. In Phase 2, out of 173 participants who completed the Colour Identification task, we excluded five participants who did not provide sex assigned at birth, and three participants who scored lower than 80% accuracy. Across all Phase 2 trials and participants, 22 trials had RT lower than the 200 ms cutoff and were excluded. Data from a total of 427 participants (182 autistic, 245 controls) were analysed.

The average RT for colour identification was 431 ± 43 ms*.* A linear mixed effects regression on target RT revealed a main effect of condition (*b* = − 2.07, *t* = − 2.45, *p* = 0.042) and a main effect of autism diagnosis on RT (*b* = − 12.14, *t* = − 2.45, *p* = 0.015), with participants responding more quickly to *potential* conditions and autistic participants responding faster in general. There was no interaction between autism diagnosis and condition (*b* = 1.10, *t* = 0.69, *p* = 0.490), suggesting no group differences relating to *potential* conditions in this task. Older participants were slower to respond regardless of *potential* or *no potential* conditions (*b* = 17.27, *t* = 8.98, *p* < 0.001). There was no effect of data collection phase or of males compared to females (*b* = − 1.56, *t* = − 0.39, *p* = 0.694). There was no effect of AQ on RT, nor any interaction between AQ and potential (both *p* > 0.1) (Supplement).

A separate linear mixed effects regression with anxiety and ADHD diagnoses included as additional regressors showed no changes to the results reported above; autistic participants still responded faster in general (*b* = − 12.50, *t* = − 2.33,* p* = 0.020), with no interaction between autism diagnosis and *potential* condition (*b* = 2.20, *t* = 1.26, *p* = 0.208). There were no effects of anxiety (*b* = − 1.08, *t* = − 0.24, *p* = 0.809) or ADHD (*b* = 1.25, *t* = 0.18, *p* = 0.855) on target RT.

## Discussion

Across both our tasks, we confirmed that individuals tend to automatically “combine” two separate pieces specifically in conditions where the two pieces could potentially form a coherent, complete structure. This automatic “combining” of disjointed parts occurred significantly less during the presentation of incoherent pieces. While we found clear differences in how people perceive potential versus non-potential combinations of stimuli, we found no significant group differences in this phenomenon. This suggests that, on average, autistic and non-autistic adults similarly perceive the “Tetris effect”.

We replicated the findings from the original study [22] in the Target Detection task. We found that, on average, participants tended to false-alarm more to *potential* No Go trials compared to* no potential *No Go trials. In other words, participants more frequently confused No Go stimuli for the composite target stimuli in conditions where the No Go stimuli could potentially combine to form the target. The false alarm rate to *no potential* trials was considerably lower than that of *potential *trials, suggesting that participants had no trouble distinguishing between pieces that could not logically combine to form the target and the target itself. The different false alarm rates in *potential* and *no potential* trials confirms that this finding is due to perceptual processing rather than simply response inhibition during No Go trials. By employing a response inhibition Go/No Go paradigm with time constraints, we were able to capture the automatic and implicit nature of low-level stimulus perception. At the same time, we found no evidence of group differences between autistic and non-autistic participants in their false alarm rates to *potential *versus *no potential *stimuli. Next, we conducted a Bayesian SDT analysis to confirm these results. The SDT analysis on trial types yielded overlapping Autism and Control group distributions in the discriminability index (d′) between *potential* and *no potential *trials, suggesting no group differences in perception (Fig. 2). Thus, as we found no group differences in the false alarm rates and in the discriminability index (d′), we conclude that autistic and non-autistic participants did not differ in their implicit, low-level perception of potential wholes from disjointed parts in this task.

We subsequently investigated whether the “Tetris effect” could be seen when the stimulus shape was made irrelevant to the task at hand. In the Colour Detection task we found that, as hypothesised, individuals were faster to respond to the target when the previous trial contained a disjointed, possible version of the target (i.e. in potential conditions), again replicating the results from the original study [22]. In other words, the presentation of two shapes that could logically form a whole primed participants to respond faster to the whole target. Notably, in this particular task, participants were instructed to attend to the stimulus colour, rather than shape. By instructing participants to focus on colour, we were able to counter the possible confound of attention on shape perception. This suggests that the mental representation of logical wholes from disjointed parts has an impact on perception even when structural properties are irrelevant to the task. Similar to what was found in the Target Detection task, there were no group differences between autistic and non-autistic participants across the different stimulus conditions. Overall, as we found no group differences in our main analyses across both tasks, we conclude that autistic and non-autistic participants similarly show the automatic and implicit perception of wholes from coherent pairs of shapes.

A prominent hypothesis is that autistic individuals rely less on top-down prior knowledge [9, 36]. However, this “hypo-priors” explanation cannot be generalised to all types of stimuli. For example, the “light-from-above” prior, or the innate belief that a light source always comes from above a visual scene, was found to be the same in both autistic and non-autistic children [37]. Other examples are illusions, which serve as a convenient means of assessing the influence of priors on perception. While autistic children have been found to be comparatively less susceptible to induced auditory illusions [38], their perception of illusory contours appears to be comparable to non-autistic children [39]. This suggests that autistic people may differ in the learning of new, contextual priors, while their pre-existing "structural" priors may be intact [40–42]. While contextual priors can be explicitly learned (e.g., knowing how to respond to social cues based on context or prior interpersonal interactions), structural priors encompass intuitive beliefs about the world (e.g., predicting how a shadow falls based on the assumption that the sun is above) [4, 43]. Accordingly, we surmise that the automatic and intuitive perception of composite structures from disjointed parts requires reliance on structural priors, thus leading to similar findings in autistic and non-autistic participants.

These findings highlight an aspect of cognition where autistic people perform similarly to non-autistic people. Although understanding cognitive difficulties in neurodivergence is fundamental in supporting autistic people to function well in their lives, likewise, it is important to explore areas where autistic people are competent and/or skilled. This strengths-based approach is increasingly employed in therapies, with the use of strengths strongly predicting better quality of life, subjective well-being, and lower levels of anxiety, depression, and stress in autistic people [44]. If autistic people have a similar drive to non-autistic people for perceptual cohesion of these block shapes, as our findings suggest, this could help explain why play-therapies and communities based on the use of e.g., Lego and Minecraft are being shown to help with social communication, engagement, and mental well-being in the autistic community [20, 45–47].

In the Colour Identification task, we found faster RTs in the autistic group compared to controls. Previous research on whether autistic people show slower response times is mixed and nuanced. A recent meta-analysis has concluded that autistic people are slower to respond across three different categories of task—measuring simple reaction time, choice reaction time, CRT (like the Colour Identification task used here), and interference control time [48]. However, the authors state the limitation that the CRT analysis was based on only 9 studies. An earlier analysis that included 23 CRT studies concluded that overall there was no difference in RTs between autistic and non-autistic people [49]. The existence and direction of effects also appear to be context- and stimulus-dependent; in certain tasks, such as the embedded figures task [14, 50, 51] and the block design test [15, 52]; it is well-established that autistic people tend to respond faster than controls. This difference has also been found in visual search tasks, where participants look for specific target [13, 53]. Here, we align our finding of faster responses in autistic individuals with these latter studies.

## Limitations

Although power calculations were carried out to assess sample sizes needed to detect a group difference, given the small effect size (Cohen’s d = 0.37) in the original study [22], it may be that any existing group differences are still undetectable with the current sample size. However, in the interest of reproducible science, we believe that such a small (undetectable) difference would likely not be meaningful to the autistic community. Our tasks may also capture differences in local-vs-global precedence, rather than perceptual cohesion. However, due to the proximity and low number of pieces in our stimuli, we believe that local or global processing styles do not play a meaningful role in these tasks. Furthermore, as participants differed in their responses to *potential* and *no potential* stimuli conditions, this suggests that our findings are due to the “Tetris effect” or the perceptual drive for cohesion. If local-to-global precedence had an impact on task performance, participants would have been equally affected by the *potential* and *no potential* stimulus pairs. The autistic sample tested in the study consisted of 61% female participants with an average age of 41 years old, thereby precluding findings that might indicate an autism-related difference in younger adults and in a more stereotypical autistic group. We further acknowledge the imbalanced sex ratio of our study participants. At the same time, we do not consider this a concern as the higher proportion of female respondents reflects a common trend in online surveys [54]. Furthermore, the historical under-diagnosis and under-representation of autistic women in research may have shaped prior perspectives of the sex ratio [55, 56]. Participants in Phase 2 of the study were recruited via Prolific while Phase 1 participants were not. As Prolific participants are paid, we note the possible effect of monetary incentive on performance. However, as we control for study phase in our analyses, we believe that any such effect has been accounted for. Another possible confound of the study is the “gravity effect” caused by the perception of momentum based on principles of Newtonian physics [57, 58]. Despite the use of static stimuli, it is possible that the human brain uses “intuitive physics” to make physical predictions [59]. In the Target Detection task, the vertically-arranged stimuli may create a structural prior wherein the Tetris pieces are expected to “fall” into place to create a composite structure. However, the Colour Detection task is not as susceptible to gravity priors due to the inclusion of horizontally-arranged stimuli. As we found the *potential* conditions to have consistent effects across both tasks, similar to the findings of Guan and Firestone (2020) [22], we conclude that our findings are not due to possible effects of gravity priors.

## Conclusions

Overall, our findings are consistent across both tasks. In general, participants showed the “Tetris effect” by perceiving two separate parts as a composite whole in the conditions where the parts could potentially combine to create a coherent whole. This effect was confirmed through two main findings: 1) participants showed higher false alarms to separate pieces that had the potential to form the composite target, suggesting that they were confusing the disjointed pieces for the target, and 2) participants responded faster to targets when the preceding stimulus consisted of separate pieces that had the potential to form the composite targets. Accompanying this, we found no group differences between autistic and non-autistic participants in stimulus-relevant false-alarm rates and response times across both tasks. Future studies employing neuroimaging methods may provide deeper insights into the neural mechanisms underlying these findings, offering a more comprehensive understanding of the processes involved. We conclude that the “Tetris effect” has a strong influence on perception and can also be found in neurodiverse populations. Our findings challenge the deficit-focussed narrative often seen in the autism literature and highlight the similarities in task performance between autistic and non-autistic participants.

## Supplementary Information

Additional file 1.

## Abbreviations

AQ: Autism spectrum quotient
ADHD: Attention deficit hyperactivity disorder
CRT: Choice reaction time
RT: Reaction time
SDT: Signal detection theory
STAI: State-trait anxiety inventory

## Declarations

### Availability of data and materials

The datasets generated and analysed during the current study are available in the OSF repository, https://osf.io/h9sjv/. Plans to replicate Guan and Firestone (2020) and investigate similar research questions in autistic and non-autistic adults were pre-registered (10.17605/OSF.IO/2QVB5).
