# Supplementary material for: The “Tetris effect”: autistic and non-autistic people share an implicit drive for perceptual cohesion
Source: Mol Autism. 2025 Mar 26;16:22. doi: 10.1186/s13229-025-00654-4 (PMC11948850; doi:10.1186/s13229-025-00654-4)
Supplement: Supplementary file 1 — Additional file 1. [file 13229_2025_654_MOESM1_ESM.pdf]

Supplementary Material

The "Tetris effect": Autistic and non-autistic people share an implicit drive for perceptual cohesion

Nazia Jassim*, Brónagh McCoy*, Esther Wing-Chi Yip, Carrie Allison, Simon Baron-Cohen, Rebecca P. Lawson

Questionnaires

A longer version of the Autism Quotient questionnaire (AQ-50) was used in Phase 2 to address questions out of the scope of this manuscript, and the equivalent AQ-10 questions were extracted and scored accordingly, to align with Phase 1 (a similar approach has been used in previous research [1]). AQ items are answered on a 4-point Likert-scale: definitely agree, slightly agree, slightly disagree, definitely disagree. The original scoring scheme is binary [2], which doesn't consider the level of agreement or disagreement with each statement. Studies since then have shown greater reliability by including all four levels in scoring [3,4]. Here we used extended scoring, which resulted in a minimum total score of 10 (no autistic traits) and a maximum score of 40 (full range of autistic traits). The STAI questionnaire was scored in the trait domain only (STAI-T) and standard scoring was applied, with scores ranging from 20-80 [5].

In Phase 1, out of the 296 participants who participated, 42 participants completed the behavioural tasks but did not complete the AQ or STAI questionnaires. The analyses incorporating autistic or anxious traits therefore do not include these participants. All participants completed the questionnaires in Phase 2.

Group scores on the AQ-10 were 32.30±4.24 in the autistic group and 21.93±4.82 in controls (see Figure S1), yielding a large group difference ($t(408)=23.22$, $p<.001$). Autistic participants also scored significantly higher on the STAI-T ($t(407)=6.74$, $p<.001$; Autism: 54.69±12.32, Control: 46.05±13.67).

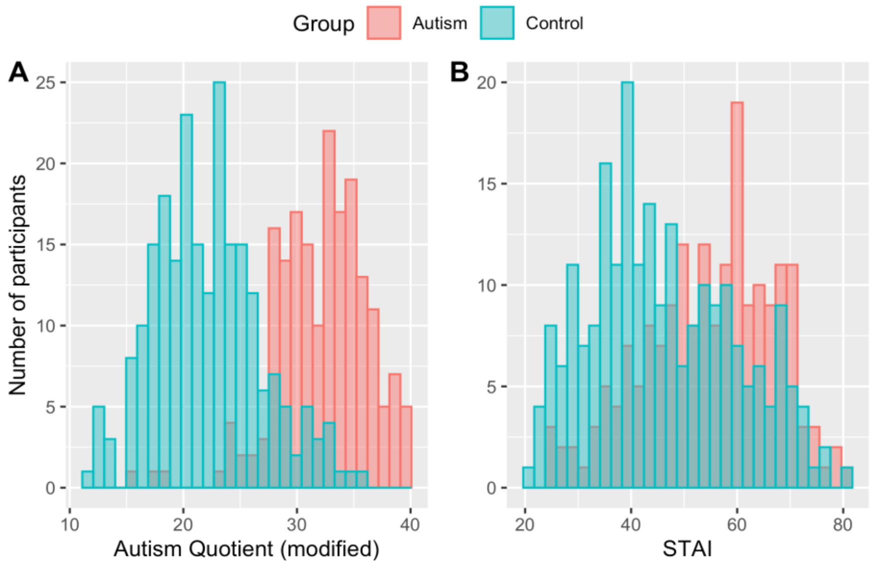

Fig. S1 *A) Distribution of AQ-10 scores, with extended scoring, and B) Distribution of STAI trait scores.*

Task 1: Target Detection

The false alarm rate for No Go trials in Phase 1 was 0.71±3.13% and 1.28±2.92% in Phase 2. A Welch's two-sample t-test indicated that the difference in false alarm rate across the two phases was significant, with significantly more false alarms in Phase 2 (t(736) = 2.78, p=.006). This difference is accounted for in the logistic regression models, using data collection phase as an independent variable. We also tested for the effects of autistic traits (as measured by the AQ-10) and anxiety-related traits (as measured by STAI) on false alarms. Separate linear mixed effects models showed that there were no main effects of AQ scores (b=-0.04, z=-0.17, p=.86) or STAI scores (b=0.03, z=0.16, p=.88) on false alarm rate, nor any interaction of No Go *potential* with AQ (b=0.12, z=0.64, p=0.52) or STAI scores (b=-0.06, z=-0.37, p=0.71).

For the hierarchical Bayesian SDT analysis, the *potential* trials were considered the "signal" and the *no potential* trials the "noise". The discriminability index or the sensitivity (d′) to the difference between *potential* trials (signal) and *no potential* trials (noise) was calculated using the following equation:

$$d' = Z(Potential\ Response\ Rate) - Z(No\ Potential\ Response\ Rate)$$

The SDT model also included a second parameter, the decision criterion (c), or "bias" in responding to *potential* (signal) versus *no potential* (noise), which was calculated as follows:

$$c = \frac{-[Z(Potential\ Response\ Rate)\ +\ Z(No\ Potential\ Response\ Rate)]}{2}$$

where Z(.) is the inverse of the cumulative distribution function of the given Gaussian distribution. While d' captures the difference in perceptual sensitivity to potential versus no potential trials, *c* is representative of a general decision or strategy bias, i.e., the general willingness to indicate that the signal is present. Our primary parameter of interest was d' but here we include results for *c* to provide full information on the model output (see Figure S2).

To obtain group differences in the discriminability index (d′), the Autism group-level posterior distribution of each parameter was subtracted from its corresponding Control group posterior, and the highest density interval (HDI) of the difference was assessed; if the interval excluded 0 then it denoted a group difference in that parameter. All Bayesian models were run with 3 chains for 4000 samples, with the first 2000 samples discarded as burn-in. Model fits and convergence were assessed using Gelman-Rubin r-hat statistics on all parameters and visual inspection of the sampling chains to

ensure they moved freely around the parameter space. Both models (one for each group) showed good fits with no convergence issues. Gelman Rubin R-hat statistics for all parameters were below the acceptable threshold of 1.1. Posterior predictive checks (PPCs) were carried out to assess that the fitted model was representative of the observed data (see Figure S3).

To assess for any effects of autistic or anxious traits on the d' or c parameters of the SDT model per group, individual-level parameters were used in separate linear regression models with d' or c as the dependent variable, and AQ, STAI, age and sex as independent variables. There were no effects of autistic traits on d' (ASC: b=-0.002, p=0.31; NT: b=0.002, p=.51) or c (ASC: b=0.009, p=0.72; NT: b=-0.003, p=.91), nor any effects of anxious traits on d' (ASC: b=-.001, p=0.84; NT = b=-.004, p=0.21) or c (ASC: b=.009, p=0.72; NT: b=0.006, p=0.81).

Both groups were equally sensitive to the difference between *potential* and *no potential* trials, as assessed by the discriminability index. We found a minor group difference in the decision criterion (c), with the Autism group showing evidence of a slightly higher decision criterion compared to the Control group (Autism: 2.9±0.11; Control: 2.65±0.07; 95% HDI = [0.004, 0.514] (Figure S2). However, we interpret this small group difference in decision criteria with caution as the task does not measure explicit cognitive processes or strategy, and the difference was not consistent across Phase 1 and Phase 2 datasets (see Figures S4 and S5).

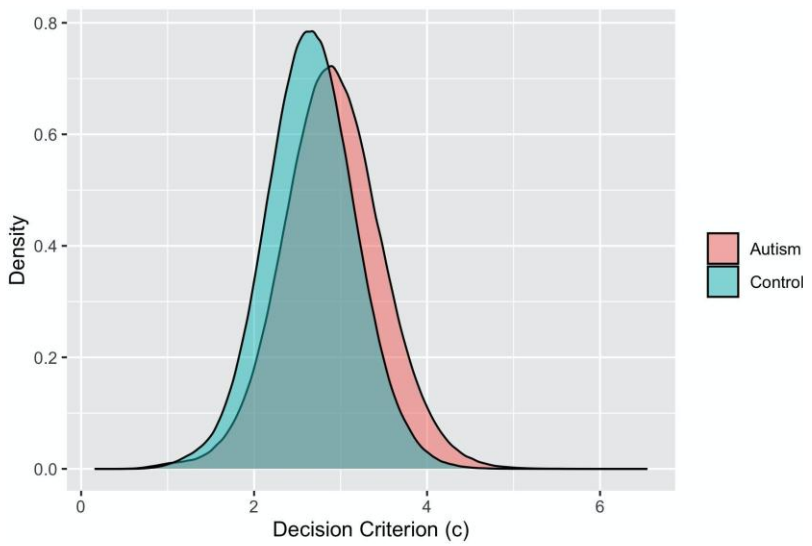

Fig. S2. *SDT analysis results showing posterior distributions of the decision criterion (c) values for Autism (in red) and Control (in blue) groups.*

A) B)

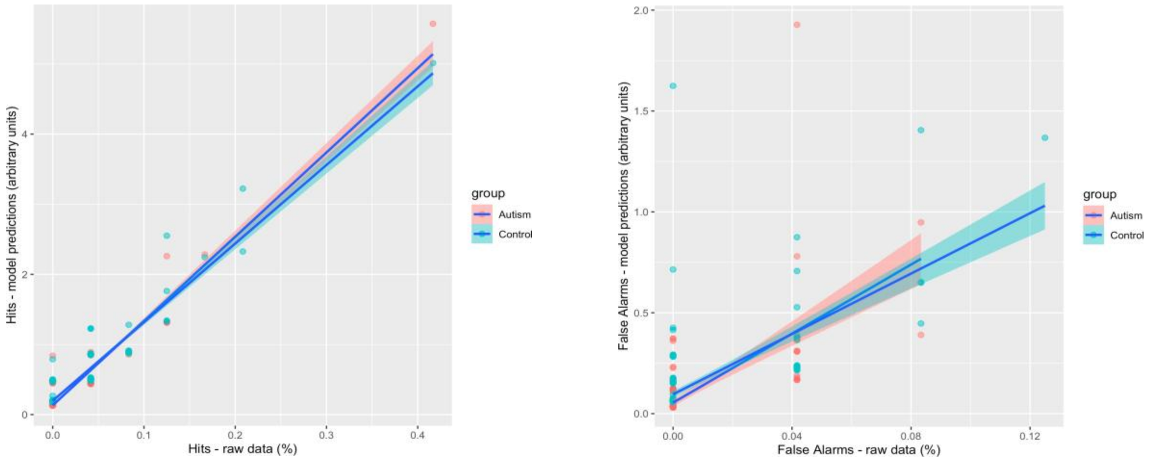

Fig. S3. Posterior Predictive Checks (PPC) on the SDT model. **A)** Comparing “Hits” (responding to Potential trials) of original data to model predictions (arbitrary units). **B)** Comparing” False Alarms” (responding to No Potential) of original data to model predictions. Hits and False Alarms are both highly and similarly correlated in each group (Hits: r = 0.97, p<.001; False Alarms: r=0.66, p<.001). This model was set up as “signal” for Potential trials and “noise” for No Potential trials, with Hits representing responses to Potential trials. However, to note, both Potential and No Potential trials are NoGo trials. With this model we are simply addressing the difference in responses to Potential vs No Potential trials.

For full transparency, SDT models were also plotted separately for each Phase 1 and Phase 2 dataset (Figures S4 and S5). Overlapping distributions again demonstrate no group-level differences in model parameters.

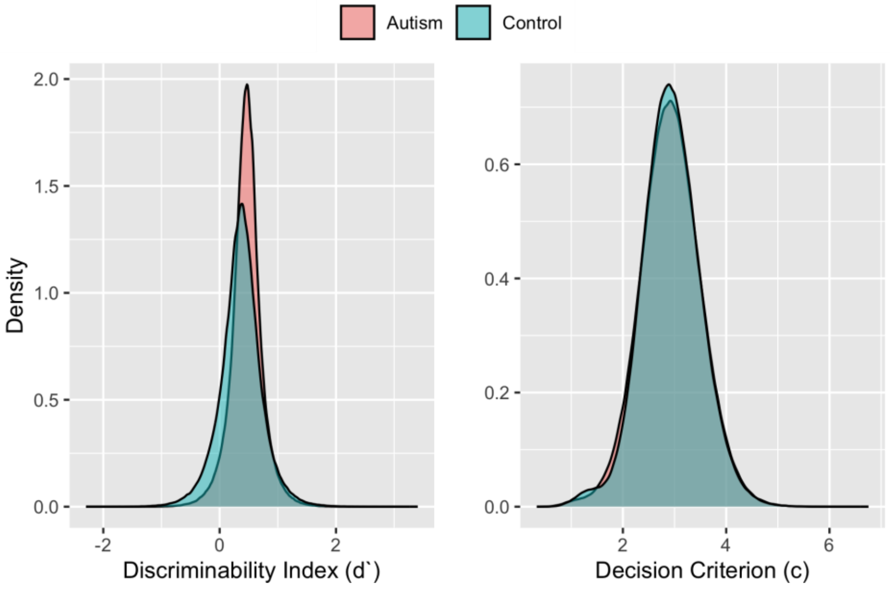

Fig. S4. *Phase 1 dataset: Distributions of the signal detection discriminability index (d’) for and decision criterion (c) values. Note: The Phase 1 dataset is made up of 180 autistic participants (in red) and 107 non-autistic participants (in blue).*

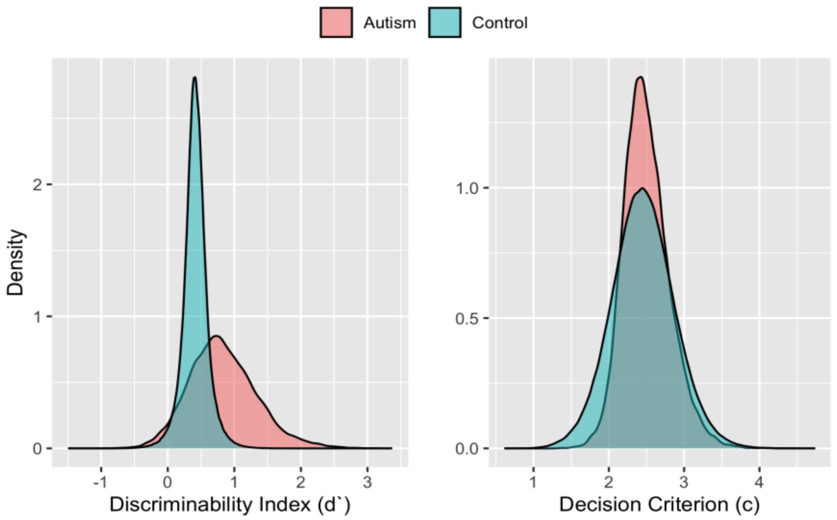

Fig. S5. *Phase 2 dataset: Distributions of the signal detection discriminability index (d') for and decision criterion (c) values. Note: The Phase 2 dataset is made up of 8 autistic participants (in red) and 159 non-autistic participants (in blue).*

Finally, to provide a comprehensive overview of performance in Go versus No-Go performance, i.e., hit and miss rates in target detection that do not relate to potentiality, we fit all data in a separate hierarchical Bayesian SDT analysis, where *Go* trials were considered the "signal" and *No-Go* (e.g., potential, no potential, and neutral) trials were considered the "noise". Discriminability index posterior distributions were similar for both groups (HDI overlapped 0), showing that both groups were similarly sensitive to the difference between *Go* and *No-Go* trials. There was a group difference in the decision criterion (*c*), with the Autism group demonstrating a higher decision criterion compared to the Control group (Autism: 0.44±0.05; Control: 0.22± 0.04; 95% HDI = [0.103, 0.348] (Figure S6). This difference in decision criterion across all trials suggests a general bias in autistic participants to indicate that the signal is present.

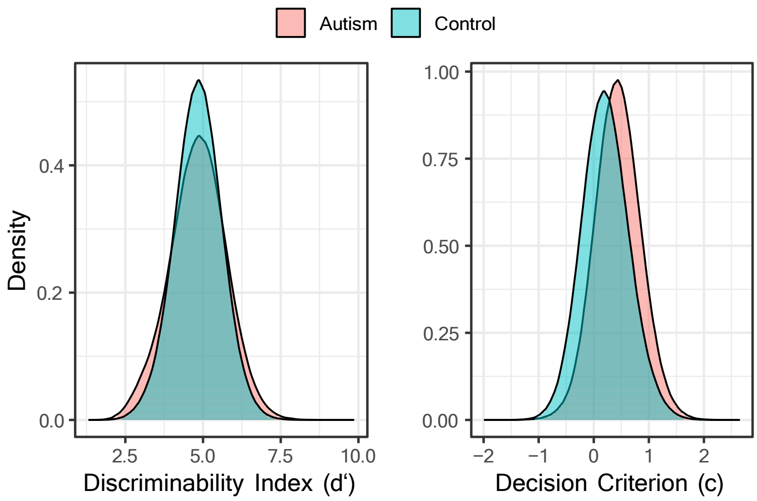

Fig. S6. *Go vs. No-Go on all trials: Posterior distributions of the signal detection discriminability index (d') and decision criterion (c).*

Task 2: Colour Identification

The average RT for correct target trials was 431±43ms (Phase 1: 436±47ms; Phase 2: 423±36ms), with significantly shorter RTs in Phase 2 ($t(409)=3.096$, $p=0.002$). Note that despite Phase 2 consisting mainly of control participants, by taking data collection phase into account in the regression models, we found that autistic people (mainly in Phase 1) were in fact faster to respond compared to controls. Similar to the Target Detection task, we also tested for the effects of autistic and anxious traits on RT to correct targets. Separate linear mixed effects models showed that there was no significant main effect of AQ scores ($b=-3.66$, $t=1.48$, $p=0.141$) or STAI scores ($b=-1.93$, $t=0.93$, $p=0.352$), nor any interaction between AQ or STAI traits and potential ($p>.1$).

References

1. Ashwood KL, Gillan N, Horder J, Hayward H, Woodhouse E, McEwen FS, et al. Predicting the diagnosis of autism in adults using the Autism-Spectrum Quotient (AQ) questionnaire. Psychol Med. 2016;46(12):2595–604.

2. Baron-Cohen S, Wheelwright S, Skinner R, Martin J, Clubley E. The autism-spectrum quotient (AQ): evidence from Asperger syndrome/high-functioning autism, males and females, scientists and mathematicians. J Autism Dev Disord. 2001;31(1):5–17.

3. De Groot K, Van Strien JW. Self-Report and Brain Indicators of Impaired Emotion Regulation in the Broad Autism Spectrum. J Autism Dev Disord. 2017;47(7):2138–52.

4. Murray AL, Booth T, McKenzie K, Kuenssberg R. What range of trait levels can the Autism-Spectrum Quotient (AQ) measure reliably? An item response theory analysis. Psychol Assess. 2016;28(6):673–83.

5. Spielberger CD. State-trait anxiety inventory for adults. 1 APA PsycTests . 1983; https://doi.org/10.1037/t06496-000. Accessed 15 Jan 2025.
